# Impact of Adverse Childhood Experiences (ACEs) on Mental Health Help-Seeking Among Asian American Adults: Findings from the 2021 California Health Interview Survey

**DOI:** 10.1007/s10903-026-01874-3

**Published:** 2026-02-18

**Authors:** Cindy Chwa, Biblia S. Cha, Gloria J. Kim, Judith Borghouts, Elizabeth Eikey, Dana B. Mukamel, Stephen M. Schueller, Margaret Schneider, Nicole A. Stadnick, Kai Zheng, Dara H. Sorkin

**Affiliations:** 1 Department of Medicine, University of California, Irvine, Irvine, USA; 2 Herbert Wertheim School of Public Health and Human Longevity Science; and The Design Lab, University of California, San Diego, San Diego, USA; 3 Department of Psychological Science; and Department of Informatics, University of California, Irvine, Irvine, USA; 4 Department of Population Health and Disease Prevention, University of California, Irvine, Irvine, USA; 5 Department of Psychiatry; Altman Clinical and Translational Research Institute Dissemination and Implementation Science Center; and Child and Adolescent Services Research Center, University of California, San Diego, San Diego, USA; 6 Department of Informatics, University of California, Irvine, Irvine, USA

**Keywords:** Adverse childhood experiences (ACEs), Asican Americans, Mental health help-seeking, Psychological distress, California health interview survey (CHIS)

## Abstract

Cumulative adverse childhood experiences (ACEs) and psychological distress have been shown to negatively affect mental health across the lifespan. Less is known, however, about how ACEs might impact mental health help-seeking behavior in adulthood, especially among Asian Americans, a population with high exposure to trauma who also face cultural barriers to mental health care. The study’s aim was to evaluate the prevalence of ACEs within a sample of Asian American respondents, the relationship of ACEs and psychological distress with help-seeking, and the association of ACEs with different types of help-seeking behaviors. Data from the 2021 California Health Interview Survey (N = 4,345) were analyzed. Pairwise comparisons examined differences in ACEs and covariates across the seven Asian American subgroups (Chinese, Filipino, Japanese, Korean, Vietnamese, South Asian, and Other Asian American). Multivariable logistic regression analyses evaluated the relationship between ACEs and covariates with professional and emerging digital mental health help-seeking behaviors. The joint effect between ACEs and Asian American subgroup was evaluated for each type of mental health help-seeking. Covariates included psychological distress, gender, age, marital status, insurance, education, English proficiency, self-rated health, and being U.S.-born. Asian American adults with 4 + ACEs were more likely to seek mental health help from primary care practitioners, mental health professionals, and social media/blogs/online forums than respondents with ≤ 3 ACEs. Moderate/severe psychological distress increased likelihood to seek mental health help. No significant interaction between ACEs and Asian American subgroup was found. Findings indicate that Asian American respondents with elevated ACEs and distress are more likely to seek mental health help from professional and emerging digital resources. This suggests a demand for these resources among those with higher needs. Differences across ACEs and distress levels in help-seeking behaviors emphasize the need for culturally tailored interventions and accessible mental health resources to better support the diverse help-seeking preferences within the Asian American community.

## Introduction

Adverse childhood experiences (ACEs), defined by stressful or traumatic events experienced before age 18, was first coined in the 1998 Adverse Childhood Experiences study [1]. ACEs encompass a range of childhood traumas, including violence, maltreatment, and serious household dysfunction. Asian Americans, one of the fastest-growing U.S. racial/ethnic groups [2], have been reported to have lower ACEs scores compared to White, Black, Hispanic, and Native American counterparts [3]. However, research indicates acculturation and migration-related stressors can contribute to psychological distress, depression, anxiety, and suicidal ideation among Asian Americans [4, 5], and further studies have found that acculturation-related stress moderates the relationship between ACEs and depressive symptoms [6]. Given the vast heterogeneity within the Asian American population, disaggregating data by subgroup is essential for accurately capturing these nuances in culturally relevant research [7–9]. Prior research indicates that Asian American subgroups experience diverse contexts, including differences in immigration history, cultural practices, socioeconomic status, education, and employment factors, which shape their immigration and settlement. For example, Filipino Americans immigrated from the Philippines as a result of imperialism and colonization from the U.S. and Spain [10], while Vietnamese, Laotian, Hmong, and Cambodian refugees immigrated to the U.S. fleeing war and violence, and commonly faced educational and occupational attainment challenges upon arrival [11, 12]. Furthermore, Korean Americans immigrated over the last century in waves as laborers, war brides, orphans, international adoptees, students, and white-collar professionals [13, 14].

In response to the exposure of many immigrants to adverse experiences and stressors related to migration, some researchers have sought to expand the ACEs framework to include adverse effects related to migration [15, 16] as well as psychological violence from racialized immigration policies, immigration enforcement, and anti-immigrant rhetoric [17]. Furthermore, studies have found historical trauma (the experience of collective trauma among a population [18]) is largely associated with intergenerational negative health and mental health impacts [19–21]. Intergenerational trauma refers to transmission of trauma or its legacy from one generation to the next, affecting psychological well-being and behavior. Among Asian Americans, examples include descendants of Japanese Americans subject to internment during World War II [22], or children of refugees who experienced and fled war, genocide, or discrimination in Cambodia [23, 24]. Studies have also found ACEs vary by generation among Asian Americans [25], in part due to conflicting cultural differences between first and second generation immigrants [26, 27].

To further understand the impact of ACEs, it is crucial to consider factors whose effects become more pronounced as the number of ACEs increases, such as psychological distress [28, 29]. Psychological distress, a “state of emotional suffering” marked by anxiety, depressive, and somatic symptoms, is a critical marker for mental health outcomes and a pathway through which adverse experiences shape lifelong well-being [30]. Psychological distress has been shown to vary by Asian American subgroup, influenced by various factors, such as poverty, working status, social cohesion, English proficiency, and acculturation [31]. Studies suggest ACEs may increase the risk of psychological distress [32], including among Asian Americans [33], potentially amplifying the negative impacts of psychological distress. These findings underscore the importance of considering distress when examining impacts of ACEs.

### ACEs, Psychological Distress, and Mental Health Help-Seeking

The relationships between psychological distress and ACEs and mental health help-seeking are complex [28, 29]. Existing literature has shown mixed findings regarding ACEs’ association with help-seeking, including varying sources of professional (e.g., primary care physicians, psychiatrists) and informal (e.g., friends, family) mental health support [34]. In the general U.S. population, some studies have concluded ACEs have no association with physician checkups but higher emergency department use [35], while others conclude a negative association with physician checkups [36]. Other studies among college students suggest that more ACEs exposure is associated with both informal and professional help-seeking [37], or positively associated with formal, but not informal, help-seeking [38]. However, there is a dearth of research examining how experiences with ACEs are associated with help-seeking among Asian Americans, including specific subgroups.

Similarly, studies have found the association between psychological distress and help-seeking are mixed. A study based in the U.K. concluded that adults with mild-to-moderate distress preferred informal sources of help, while those experiencing severe distress preferred professional therapy [39]. On the other hand, a study among rural adults in the U.S. found that those with higher distress tended to be unwilling to seek mental health care [40]. Studies among Asian Americans indicate that various subgroups differ in their preferred sources of help (i.e. medical professionals, mental health professionals, friends, family, or non-healthcare professionals like religious clergy) [41]. Research on specific subgroups shows that Chinese Americans with higher mental health distress are more likely than lower mental distress counterparts to seek help from both informal and formal sources of mental health help, especially medical doctors [42]. Furthermore, although older adults who are Korean Americans are more likely to report psychological distress than non-Hispanic White counterparts, Japanese Americans are less likely to report distress, yet both are less likely to use mental health services [43]. Research also indicates that among Asian Americans, structural factors and cues to action vary the likelihood of help-seeking by subgroup. For example, a positive social network increases the likelihood of using formal support services among Chinese and Filipinx, while structural factors like lacking ethnic concordant providers and access to healthcare hinder help-seeking for Koreans and Vietnamese [41]. In addition, Asian Americans reporting psychological distress often do not seek help for their mental health due to cultural and systemic barriers such as low perceived need, preference for informal support, and prioritizing academic or career concerns over psychological issues [41, 43–46]. A study among Asian American adolescents also highlights that many have no one to confide in [47].

In addition to traditional help-seeking, digital mental health tools have emerged as potentially effective feasible, and accessible options for providing remotely-delivered interventions to both adults [48–51] and children [52]. For example, a study among adults across the 50 states in the U.S. indicated these tools showed promise in reducing mental health symptoms [53] (including among adults with serious mental illness [51]), with an additional study drawing similar conclusions among adolescents aged 13–17 in New Zealand [54]. Two recent systematic reviews have concluded that digital mental health technology use is associated with beneficial effects related to anxiety and depression symptom severity among college students [55, 56], though an earlier one found that reach and uptake is not sufficiently reported among this population [57]. In the present study, digital mental health resources considered include online tools (such as mobile apps and texting services), seeking mental health-related support by connecting with others through social media, blogs, and online forums, as well as using social media to do hashtag searches or follow people with similar conditions. These tools may be particularly beneficial for those experiencing ACEs [58, 59]. However, there is limited scholarship on Asian Americans’ engagement with digital mental health resources as a form of help-seeking, and their specific needs and experiences concerning digital mental health tools and interventions [60].

### The Current Study

This study examined ACEs and help-seeking (via professional and emerging digital mental health resources) in disaggregated Asian American subgroups, considering the influence of distinct socio-demographic experiences. Using an ethnically and linguistically diverse sample, the aims of the study were to: (1) examine the frequency of reported ACEs across Asian American subgroups; (2) explore the relationship that ACEs has with mental health help-seeking behaviors after adjusting for various covariates, including psychological distress; and (3) evaluate whether the relationship between ACEs and the various types of mental health help-seeking vary among respondents from diverse Asian American subgroups. Based on the existing literature, we hypothesized that:

1. Frequency of reported ACEs would be highest in respondents reporting as Other Asian or Vietnamese due to their turbulent migration histories;
2. Greater frequency of reported ACEs would be directly associated with more mental health help-seeking after adjusting for covariates; and
3. The relationship between ACEs and types of mental health help-seeking would vary by Asian American subgroup.

## Methods

### Participants and Data Collection

This study utilized 2021 data from the California Health Interview Survey (CHIS), a population-based health survey of California residents [61]. The analysis focused on the adult subsample identifying as Asian American. The survey was administered in multiple languages including English, Spanish, Chinese (Cantonese and Mandarin), Korean, Vietnamese, and Tagalog.

### Measures

#### Asian American Subgroups

Asian American subgroup was self-reported (Chinese, Filipino, Japanese, Korean, Vietnamese, South Asian, and Other Asian). South Asian included those identifying as Bangladeshi, Pakistani, Sri Lankan, Indian, Nepalese, or South Asian, or with origins from related countries. Other Asian Americans included Southeast Asian, multi-Asian identities, and subgroup sample sizes under 250.

#### Adverse Childhood Experiences (ACEs)

In the CHIS, ACEs were measured using the 2009–2020 Behavioral Risk Factor Surveillance System’s ACE Module [62]. This scale contains 11 items addressing eight domains: household mental illness, household substance misuse, family member incarceration, parental separation or divorce, parent treated violently, child physical abuse, child emotional abuse, child sexual abuse. We dichotomized scores into “ ≤ 3 ACEs” and “4 + ” ACEs, because research has linked 4 + ACEs to adverse health and life outcomes [63, 64].

#### Mental Health Help-Seeking from Healthcare Professionals

Mental health help-seeking from primary care practitioners was assessed by: “In the past 12 months have you seen your primary care physician or general practitioner for problems with your mental health, emotions, nerves, or your use of alcohol or drugs?” with responses “Yes/No.” Mental health help-seeking from mental health professionals was assessed by: “In the past 12 months have you seen any other professional, such as a counselor, psychiatrist, or social worker for problems with your mental health, emotions, nerves, or your use of alcohol or drugs?” with responses “Yes/No.”

#### Mental Health Help-Seeking Through Emerging Digital Mental Health Resources

Mental health help-seeking through mobile apps and texting services was assessed by: “In the past 12 months, have you tried to get help from an on-line tool, including mobile apps or texting services for problems with your mental health, emotions, nerves, or your use of alcohol or drugs?” with responses “Yes/No.” Mental health help-seeking from similar others (i.e., peers) by connecting through social media, blogs, and online forums was assessed by: “In the past 12 months, have you connected online with people that have mental health or alcohol/drug concerns similar to yours through methods such as social media, blogs, and online forums? Include online forums or closed social media groups on specific issues, doing hashtag searches on social media, or following people with similar health conditions.” with responses “Yes/No.”

#### Covariates

Sociodemographic variables included the following: gender (female or male), age, marital status (currently married or not currently married), insurance status (currently insured or not currently insured), highest education (high school or less, some college, or some graduate school), English proficiency (yes or no), self-rated health (good/excellent or fair/poor), and born in the U.S. (yes or no). In CHIS, psychological distress was also included as a covariate and measured using the Kessler Psychological Distress Scale (K6) [65]. A two-level categorical variable was created, with none/low distress as sum scores between 0–4 and moderate/severe distress as scores equal to or greater than 5. The cutoff of K6 ≥ 5 was the optimal lower threshold cut-point indicative of moderate mental distress based on a 2007 CHIS validity study [66].

### Analysis

Descriptive statistics were calculated to describe survey respondents’ demographics. We assessed differences across ethnicities using Chi-Squared tests. Statistical significance was set at 2-sided *P* = 0.05. Four multivariable logistic regression models were fit examining ACEs and each of the four mental health help-seeking outcomes, adjusting for all covariates. Chinese was the reference group for ethnicity due to having the largest sub-sample size. Finally, four multivariable logistic regression models examined the interaction of ACEs and Asian American subgroups, adjusting for all covariates. All analyses were conducted using SAS Version 9.4 (SAS Institute Inc., Cary, NC). This study did not require additional approval from an institutional and/or national ethical review committee as data was deidentified prior to analysis.

## Results

Table 1 displays overall participant demographics by ethnicity. Among 4,345 participants, 36.7% were Chinese, 14.4% Filipino, 8.3% Japanese, 10.3% Korean, 11.9% Vietnamese, 7.4% South Asian, and 11.0% Other Asian. Though there were no significant differences by gender, South Asians were significantly younger than the Chinese, Filipino, Japanese, Korean, and Vietnamese respondents. Other Asians were more likely to be married than Chinese, Filipino, Koreans, and South Asian respondents. Insurance status did not significantly vary across ethnicities. Filipino and Korean respondents reported the highest percentage of attending some college (66.4% and 57.0% respectively), while Vietnamese Americans reported the lowest (39.5%). Most respondents reported speaking English proficiently, with South Asians most likely (99.1%) and Vietnamese least likely (62.5%). Most reported good/excellent health, with Japanese reporting the highest (91.1%) and Vietnamese the lowest quality of health (70.8%). South Asians were most likely to have been born outside the U.S. (75.0%), while Japanese were most likely to have been born in the U.S. (69.1%). The prevalence of 4 + ACEs varied, with highest rates among Other Asian (19.5%), followed by Japanese (16.0%), Korean (14.0%), Vietnamese and South Asian (both 11.6%), Filipino (10.5%), and Chinese (8.8%). Koreans (57.3%) and Other Asians (57.1%) reported more moderate/severe distress than the other subgroups.

**Table 1:** Participant demographics (N = 4,345) (N, %)

	Chinese (1,596, 36.7%)	Filipino (625, 14.4%)	Japanese (362, 8.3%)	Korean (446, 10.3%)	Vietnamese (516, 11.9%)	South Asian (323, 7.4%)	Other Asian (480, 11.0%)	*P* value
Gender								0.07
Female	792 (51.0)	368 (56.2)	213 (56.0)	225 (50.2)	254 (57.2)	137 (46.0)	256 (54.6)	
Male	804 (49.0)	257 (43.8)	149 (44.0)	221 (49.8)	262 (42.8)	186 (54.0)	224 (45.4)	
Age, *years* (mean) (SD)	45.3 (0.73)^a^	45.3 (1.04)^b^	47.9 (1.45)^c,d^	45.1 (1.22)^e^	47.1 (1.29)^f^	39.6 (1.00)^a,b,d,e,f^	42.6 (1.41)^c^	** < 0.01**
Marital Status								**0.03**
Not married	641 (42.6)^a^	249 (45.3)^b^	183 (49.3)	150 (41.4)^c^	228 (45.9)	129 (40.0)^d^	245 (54.6)^a,b,c,d^	
Married	955 (57.4)	376 (54.7)	179 (50.7)	296 (58.6)	288 (54.1)	194 (60.0)	235 (45.4)	
Insurance Status								0.23
Insured	1536 (93.6)	601 (95.2)	357 (97.9)	413 (91.7)	498 (95.4)	312 (95.8)	456 (91.2)	
Uninsured	60 (6.4)	24 (4.8)	5 (2.1)	33 (8.3)	18 (4.7)	11 (4.2)	24 (8.8)	
Education Level								** < 0.01**
High School or Less	174 (30.9)^a^	40 (14.5)^b,c^	30 (26.0)^d,e^	36 (17.5)^f,g,h^	107 (46.4)^c,e,h,j,k^	22 (15.0)^a,b,d,g,i,k^	58 (31.1)^f,i,j^	
Some college	735 (38.7)^a,b,c,d,e,f^	442 (66.4)^a,g,h,i,j^	221 (47.4)^b,g,k,l^	257 (57.0)^c,h,m^	301 (39.5)^f,l,o^	139 (40.8)^e,j,k,m,n,p^	272 (45.3)^d,i,n,o,p^	
Some grad school	687 (30.4)	143 (19.0)	111 (26.6)	153 (25.5)	108 (14.0)	162 (44.1)	150 (23.6)	
Speak English								** < 0.01**
No	238 (24.4)^a,b,c,d,e^	8 (2.7)^a,f,g,h^	16 (7.4)^b,i,j,k^	134 (26.5)^f,i,l,m,n^	119 (37.5)^e,h,k,n,p,q^	5 (0.9)^d,j,m,o,q^	39 (13.7)^c,g,l,o,p^	
Yes	1358 (75.6)	617 (97.3)	346 (92.6)	312 (73.5)	397 (62.5)	318 (99.1)	441 (86.3)	
Self-Rated Health								** < 0.01**
Poor/Fair	232 (17.5)^a,b,c^	82 (12.8)^d,e^	34 (8.9)^a,f,g,h^	82 (18.1)^f,i,j^	108 (29.2)^c,e,h,j,l,m^	33 (9.1)^b,i,k,m^	66 (18.6)^d,g,k,l^	
Good/Excellent	1364 (82.5)	543 (87.2)	328 (91.1)	364 (81.9)	408 (70.8)	290 (90.9)	414 (81.4)	
U.S. Born								** < 0.01**
No	1119 (70.8)^a,b^	400 (67.3)^c,d^	126 (34.8)^a,c,d,e,f,g^	361 (71.2)^d,h^	395 (72.0)^g,j^	250 (75.0)^f,i^	270 (48.5)^b,d,e,h,i,j^	
Yes	477 (29.2)	225 (32.7)	236 (69.1)	84 (28.8)	119 (28.0)	73 (25.0)	210 (51.5)	
ACEs								**0.04**
0–3	1461 (91.2)^a,b^	541 (89.4)^c^	313 (84.1)	381 (86.0)^a^	452 (88.8)^e^	289 (88.4)^d^	405 (80.5)^b,c,d,e^	
4 +	135 (8.8)	84 (10.5)	49 (16.0)	65 (14.0)	64 (11.6)	34 (11.6)	75 (19.5)	
Psychological Distress								
None/low distress (0–4)	943 (54.7)	372 (54.2)	221 (59.6)	212 (42.7)	316 (56.8)	184 (50.9)	251 (42.9)	** < 0.01**
Moderate/severe distress (5–24)	653 (45.3)^a,b^	253 (45.8)^c,d^	141 (40.4)^e,f^	233 (57.3)^a,c,e,g^	198 (43.2)^g,h^	139 (49.1)	229 (57.1)^b,d,f,h^	

Superscript letters (*a, b, c, *etc.) within the table denote statistically significant differences between Asian American subgroups, based on pairwise comparisons at the α = 0.05 level. Groups sharing the same letter are significantly different from each other. P-values noted in bold indicate significant differences <= 0.05

Table 2 displays the results of the multivariable logistic regression models for help-seeking. Those reporting 4 + ACEs were more likely to seek help from primary care practitioners (AOR 1.7, 95% CI 1.1–2.7; P = 0.02), mental health professionals (AOR = 1.7, 95% CI 1.2–2.5; P < 0.01), and through social media (AOR = 2.0; 95% CI, 1.2–3.2; P < 0.01) than those reporting ≤ 3 ACEs. Compared to Chinese respondents, Filipinos were more likely to seek help from a primary care practitioner (AOR = 2.6, 95% CI 1.4–5.0; *P* < 0.01), mental health professionals (AOR = 1.6, 95% CI 1.0–2.6; *P* = 0.04), and social media (AOR = 1.9, 95% CI 1.0–3.3; *P* = 0.03). In contrast, Vietnamese (AOR = 0.4, 95% CI 0.2–0.8; *P* < 0.01) were less likely to seek mental health professional help compared to Chinese respondents.

**Table 2:** Impact of ACEs on receipt of mental health help-seeking (N = 4,345)

	Primary Care Practitioner		Mental Health Professional		App/Texting		Social Media/Blog/Online Forum	
	Adjusted OR (95% CI)	*P* value	Adjusted OR (95% CI)	*P* value	Adjusted OR (95% CI)	*P* value	Adjusted OR (95% CI)	*P* value
***Main Effects***								
ACEs								
0–3	Reference		Reference		Reference		Reference	
4 +	1.7 (1.1, 2.7)	**0.02**	1.7 (1.2, 2.5)	** < 0.01**	1.5 (1.0, 2.2)	0.07	2.0 (1.2, 3.2)	** < 0.01**
Asian American Subgroup								
Chinese	Reference		Reference		Reference		Reference	
Filipino	2.6 (1.4, 5.0)	** < 0.01**	1.6 (1.0, 2.6)	**0.04**	1.4 (0.8, 2.3)	0.20	1.9 (1.0, 3.3)	**0.03**
Japanese	1.6 (0.8, 3.4)	0.19	1.3 (0.8, 2.1)	0.31	0.7 (0.4, 1.3)	0.30	1.3 (0.6, 2.9)	0.56
Korean	1.0 (0.5, 2.0)	1.00	0.8 (0.4, 1.5)	0.48	1.1 (0.5, 2.2)	0.88	0.9 (0.3, 3.0)	0.90
Vietnamese	1.1 (0.5, 2.5)	0.81	0.4 (0.2, 0.8)	** < 0.01**	0.8 (0.4, 1.6)	0.52	1.7 (0.7, 3.9)	0.21
South Asian	1.1 (0.5, 2.4)	0.86	0.8 (0.4, 1.6)	0.53	0.6 (0.3, 1.1)	0.11	0.8 (0.3, 2.6)	0.75
Other Asian	1.8 (1.0, 3.2)	0.06	1.2 (0.7, 2.0)	0.47	1.1 (0.5, 2.1)	0.87	1.2 (0.5, 2.6)	0.67
*Covariates*								
Psychological Distress								
None/Low (0–4)	Reference		Reference		Reference		Reference	
Moderate/Severe (5–24)	5.7 (3.5, 9.3)	** < 0.01**	4.8 (3.2, 7.0)	** < 0.01**	3.9 (2.6, 5.8)	** < 0.01**	3.8 (2.2, 6.5)	** < 0.01**
Age, *years* (mean)	1.0 (0.99, 1.0)	0.56	0.99 (0.98, 1.0)	0.48	0.98 (0.9, 1.0)	**0.03**	0.98 (0.95, 1.0)	0.11
Marital Status								
Not married	Reference		Reference		Reference		Reference	
Married	0.7 (0.5, 1.2)	0.19	0.7 (0.5, 1.0)	0.09	0.8 (0.5, 1.1)	0.19	1.0 (0.6, 2.0)	0.89
Education Level								
High School or less	Reference		Reference		Reference		Reference	
Some college	1.2 (0.7, 2.3)	0.48	2.4 (1.3, 4.6)	** < 0.01**	0.9 (0.5, 1.8)	0.83	0.9 (0.4, 1.9)	0.79
Some grad school	1.6 (0.8, 3.2)	0.22	4.2 (2.1, 8.4)	** < 0.01**	1.2 (0.6, 2.3)	0.54	0.9 (0.4, 2.0)	0.77
U.S. Born								
No	Reference		Reference		Reference		Reference	
Yes	1.4 (0.8, 2.6)	0.25	2.0 (1.3, 3.1)	** < 0.01**	1.2 (0.7, 1.9)	0.51	1.9 (1.0, 3.6)	0.06
Speak English								
No	Reference		Reference		Reference		Reference	
Yes	0.9 (0.4, 2.0)	0.87	0.9 (0.3, 2.5)	0.89	3.1 (1.5, 6.6)	** < 0.01**	1.8 (0.6, 5.4)	0.28
Self-Rated Health								
Fair/Poor	Reference		Reference		Reference		Reference	
Good/Excellent	0.4 (0.3, 0.7)	** < 0.01**	0.8 (0.5, 1.1)	0.17	1.0 (0.6, 1.7)	0.90	0.8 (0.5, 1.3)	0.29

^a^*P* value from the Wald test assessing whether the covariate level’s effect differs from the reference category in logistic regression

Models above have been adjusted for age, U.S. born, marital status, education level, English proficiency, self-rated health, and psychological distress. P-values noted in bold indicate significant differences <= 0.05

Compared to those experiencing none/low psychological distress, those with moderate/severe distress were more likely to seek mental health help from primary care practitioners (AOR = 5.7, 95% CI 3.5–9.3; *P* < 0.01) and mental health professionals (AOR = 4.8, 95% CI 3.2–7.0; *P* < 0.01). They were also more likely to seek help using apps/texting (AOR = 3.9, 95% CI 2.6–5.8; *P* < 0.01) and social media (AOR = 3.8, 95% CI 2.2–6.5; *P* < 0.01). Older age was associated with less help-seeking through app/texting (AOR = 0.98, 95% CI 0.9–1.0; *P* = 0.03). While there were no differences in help-seeking type by marital status, higher education levels and being U.S.-born were associated with higher likelihood of seeking help from mental health professionals. Speaking English was associated (AOR = 3.1, 95% CI 1.5–6.6; *P* < 0.01) with app/texting use, and those who reported being in good/excellent health were less likely to seek mental health help from their primary care practitioner compared to those with worse health.

There was no significant interaction between subgroup and ACEs in the prediction of help-seeking.

## Discussion

This study is among the first to disaggregate Asian American subgroups in a study of ACEs and provides a preliminary perspective on the relationship between ACEs and various types of help-seeking. Findings support some of the existing research indicating that more ACEs is associated with more help-seeking from professional sources among general populations [37, 38]. Our first hypothesis was partially confirmed, with significant ACE prevalence differences across Asian American subgroups, including nearly 20% of Other Asian reporting 4 + ACEs. Findings also confirmed the second hypothesis, as respondents reporting 4 + ACEs were more likely to seek help from primary care practitioners, mental health professionals, and through social media compared to those reporting ≤ 3 ACEs after adjusting for covariates. Our third hypothesis, however, was not supported; and the relationship between ACEs and types of mental health help-seeking did not vary by subgroup.

### Asian American Subgroups Reported Varying Frequency of ACEs

We found significant differences in ACE prevalences across the seven primary Asian American subgroups included in the study. In revealing that the “Other Asian” group reported the highest 4 + ACEs, our study aligns with significant histories of trauma and forced displacement experienced by immigrants within this group, which included respondents from Southeast Asia, such as Cambodia and Laos [23, 24]. This group also included respondents identifying with multiple Asian American subgroups, indicating multiethnic Asian identity may factor into their ACEs and mental health. Scholarship on ACEs among multiethnic Asian Americans is limited, and existing research focused on mental health of multiethnic Asian Americans tends to be focused on mixed race individuals (such as Asian White biracial individuals) with varying conclusions. Some studies indicate that Asian Americans who identify as multiracial or multiethnic have better mental health than monoethnic peers [67], while others have found multiracial Asian Americans experience more depression and anxiety than monoracial counterparts [68–70]. Furthermore, given that 52% of Other Asians were U.S.-born, intergenerational trauma, transmitted through a combination of genetic, psychological and environmental factors may have played a role [71, 72], as second-generation Asian Americans have been found to report higher ACEs than their first-generation counterparts [25]. Intergenerational trauma, coupled with cultural conflicts, acculturation stress, and unique socioeconomic pressures in the U.S., may create environments where parental stress intersects with children’s experiences, compounding ACEs risk [73].

Japanese Americans reported the second-highest ACE prevalence, a finding that may reflect both demographic factors and the lasting impact of historical trauma. While the majority of Japanese in this study were U.S.-born, which can increase the likelihood of disclosing adverse experiences [74], the intergenerational effects of Japanese internment during World War II may also play a significant role [22, 75]. This is especially noteworthy despite their positive self-rated health in this study. Chinese respondents reported the lowest ACE prevalence. The majority of Chinese respondents were foreign-born and averaged 45 years old. While they could have immigrated at any point, this age distribution aligns with historical patterns of Chinese immigration during the 1980s, a period characterized by significant migration for educational and economic opportunities in China [76]. Migration contexts can be protective against ACEs, as families immigrating for upward mobility may benefit from increased resources and family cohesion [77].

### More ACEs and Psychological Distress was Associated with Specific Types of Help-Seeking

As hypothesized, our study found that respondents reporting more ACEs were more likely to seek help for their mental health. High number of reported ACEs and moderate/severe distress were also associated with mental health help-seeking through social media/blogs/online forums, but not apps/texting. Though social media use is linked to high ACEs, psychological distress, depression, and anxiety [78, 79], it can also be protective, improving stress management and social connection among Asian Americans [59, 80, 81]. Similarly, blogs and forums have emerged as effective platforms for emotional expression and catharsis, helping individuals process trauma and reconnect with others [82, 83]. Systematic reviews highlight these tools’ potential to mitigate psychological distress, offering supportive environments that reduce isolation and foster connections [84, 85]. However, while these platforms are promising, additional research is needed to fully understand their collective role in ACE-related mental health help-seeking, particularly within Asian American communities.

The current study also found severe psychological distress was associated with seeking help from both professional healthcare sources and emerging digital resources. As Koreans (57.3%) and Other Asian Americans (57.1%) reported significantly more moderate/severe distress than the other subgroups, our findings support prior research indicating Asian American subgroups experience varying levels of psychological distress [31], which may result in subgroup-specific patterns of help-seeking. Our finding that moderate/severe psychological distress, but not ACEs, is positively associated with app/texting help-seeking aligns with prior research showing high psychological distress, including depression and anxiety, are linked to an interest in mental health apps [86]. The lack of association between ACEs and app/texting help-seeking may reflect the complex and long-term nature of ACE-related trauma, which individuals might feel requires more comprehensive, professional support [87]. In contrast, psychological distress, which can be more immediate and situational, may drive a preference for quicker, easily accessible digital solutions.

### Relationship Between ACEs and Help-Seeking Did Not Vary by Asian American Subgroup

Finally, our third hypothesis was not supported by study findings, as the relationship between ACEs and types of mental health help-seeking did not vary by subgroup. However, a non-significant interaction between ACEs and Asian American subgroups does not necessarily indicate that the psychological toll of ACEs is the same across Asian American subgroups. Rather, the current study may not have been able to detect subgroup differences due to methodological limitations, such as measurement sensitivity, sample size, and the aggregation of diverse subgroups.

### Limitations

This study has several limitations. First, its cross-sectional design limits causal conclusions. Second, ACEs were self-reported, introducing recall or social desirability biases [88]. Third, the ACEs measures do not capture acculturation or migration-related stressors in spite of these being important in Asian Americans’ life experiences. Fourth, the “Other Asian” category grouped diverse subgroups, obscuring potential subgroup-specific differences. Finally, the study findings cannot be generalized to all Asian American populations across the United States as the CHIS is a population-based health survey only of California residents.

## New Contribution to Literature

This study contributes to the literature by offering novel insights into the relationship between ACEs and various mental health help-seeking behaviors within Asian American communities. The positive association between psychological distress and help-seeking through both professional and emerging digital resources highlights the importance of addressing psychological distress to improve care access. These findings inform community-based organizations and healthcare facilities in developing targeted interventions to reduce service barriers and address disparities in mental health access, fostering more equitable care for specific Asian American subgroup populations.

## Data Availability

This data is publicly available at the UCLA Center for Health Policy Research, see https://healthpolicy.ucla.edu/our-work/california-health-interview-survey-chis
